# Prevalence and Correlates of Probable Depression and Anxiety Among Homeless Individuals During the COVID-19 Pandemic in Germany

**DOI:** 10.3390/ijerph23020154

**Published:** 2026-01-26

**Authors:** Veronika Kowalski, André Hajek, Victoria Van Rüth, Wiebke Graf, Katharina Dost, Anna Brennecke, Hans-Helmut König, Klaus Püschel, Benjamin Ondruschka, Fabian Heinrich, Franziska Stallbaum

**Affiliations:** 1 Institute of Legal Medicine, University Medical Center Hamburg-Eppendorf, 22529 Hamburg, Germanyb.ondruschka@uke.de (B.O.);; 2 Department of Health Economics and Health Services Research, University Medical Center Hamburg-Eppendorf, 20246 Hamburg, Germany; 3 Department of Medical Statistics, London School of Hygiene and Tropical Medicine, London WC1E 7HT, UK; 4 Centre for Data and Statistical Science for Health, London School of Hygiene and Tropical Medicine, London WC1E 7HT, UK; 5 I. Department of Medicine, University Medical Center Hamburg-Eppendorf, 20246 Hamburg, Germany

**Keywords:** homeless, depression, anxiety, COVID-19, mental health, public health

## Abstract

**Highlights:**

**Public health relevance—How does this work relate to a public health issue?**

- Homeless individuals constitute a vulnerable population with elevated risk for depression and anxiety, particularly during public health crises such as the COVID-19 pandemic.
- This multicentre study provides epidemiological evidence on mental health outcomes among homeless populations in Germany.

**Public health significance—Why is this work of significance to public health?**

- A considerable proportion of homeless individuals screened positive for probable depression and anxiety using validated instruments.
- Sociodemographic, behavioural, and pandemic-related factors were identified as relevant correlates of adverse mental health outcomes.

**Public health implications—What are the key implications or messages for practitioners, policy makers and/or researchers in public health?**

- Results underscore the importance of routine mental health screening within low-threshold and outreach-based services for homeless populations.
- Integrated public health strategies linking housing provision, mental health care, and substance use services are essential to reduce health inequalities.

**Abstract:**

Objective: Homeless individuals are vulnerable, and a high burden of mental health problems is suspected. We aim to identify the prevalence and key predictors of probable depression and anxiety among homeless individuals in Germany during the Coronavirus disease-2019 (COVID-19) pandemic. Study Design: Nationwide multicentre cross-sectional study including clinical, laboratory, and questionnaire-based data on the health of homeless individuals in Germany. Methods: Data were used from the *National Survey on the psychiatric and somatic health of homeless individuals* during the COVID-19 pandemic. Probable depression and anxiety were determined using the Patient Health Questionnaire 9 and the General Anxiety Disorder 2 questionnaire. Logistic regression analysis was used to identify key predictors of probable depression and anxiety. Results: A high prevalence of probable depression (26.8% [95% CI: 23.2–30.3]) and anxiety (27.2% [95% CI: 23.7–30.7]) was found. Logistic regressions showed both probable depression and anxiety associated with being female (depression: OR 1.80 [95% CI: 1.06–3.03]; anxiety: OR: 1.69 [95% CI: 1.03–2.76]), daily use of any illegal substances (depression: OR 3.20 [95% CI: 1.87–5.49]; anxiety: OR 2.04 [95% CI: 1.21–3.45]), and fear of contracting COVID-19 (little fear, depression: OR: 1.81 [95% CI 1.01–3.23], some fear, anxiety: OR: 2.52 [95% CI: 1.31–4.83]). Probable depression was associated with longer durations of homelessness (OR: 1.004, [95% CI: 1.001–1.007]. Conclusions: Probable depression and anxiety are highly prevalent in homeless individuals throughout. Key predictors may help to identify individuals in need and design targeted interventions.

## 1. Introduction

In 2022, an estimated 607,000 people were experiencing homelessness in Germany, and the numbers were shown to increase during the COVID-19 pandemic [1]. Housing is an essential factor within the social determinants of health, which the World Health Organization identified as directly influencing morbidity and mortality and contributing to health inequities and outcomes [2].

Indeed, homelessness is associated with a high prevalence of somatic diseases and mental disorders [3], as well as high rates of substance abuse and premature death [4]. Especially mental health difficulties, such as depression and anxiety, go along with several adverse health outcomes. Among others, they may contribute to homeless people frequently succumbing to drug overdose and suicide, lowering the overall life expectancy [5,6,7].

In 2017, a review of publications from the years 2000 to 2017 found mental illness requiring treatment in over 75% of the homeless population in Germany, highlighting the need for interventions aiming to reduce the mental health burden in the community of homeless individuals [8]. However, personal and structural barriers, including access to health care and medication, complicate specialised psychiatric treatment or psychologic attempts for homeless people [8,9]. During the COVID-19 pandemic, social distance policies, hygiene, and lockdown measures resulted in the deterioration of support and low threshold services for homeless people and, therefore, negatively influenced their access to health care [10]. To contextualise this study, the situation concerning COVID-19 at the time of data acquisition is explained: The 7-day incidence rate of SARS-Co-V-2 infections was around 14/100,000 inhabitants at the beginning of our study and rose to 79/100,000 inhabitants [11]. In the summer of 2021, most facilities faced difficulties maintaining regular services due to COVID-19-related restrictions [10].

Studies on depression and anxiety before the pandemic in Germany report heterogeneous prevalences with marginalised groups underrepresented [12]. Data on the prevalence of depression and anxiety in homeless people during the COVID-19 pandemic were generated from a single study in Germany, namely the *Hamburg Survey of Homeless Individuals*, a single-centre study performed in 2020. There, prevalences of probable anxiety and depression were comparable to the general population [13]. With the progression of the pandemic, the prevalence of various mental health issues has increased globally. However, the prevalence of anxiety and depression in homeless individuals in Germany has not been evaluated since then [14].

This cross-sectional multi-centre study aimed to examine the prevalence and identify key predictors of probable anxiety and depression among the homeless population in Germany in the later phase of the COVID-19 pandemic. Our data may open opportunities to identify homeless individuals at risk for anxiety and depression, benefiting from targeted intervention.

## 2. Materials & Methods

### 2.1. Sample

Cross-sectional data were used from the *National survey on the psychiatric and somatic health of homeless individuals during the COVID-19 pandemic* (NAPSHI). Data collection was performed between 26 July and 17 September 2021, in metropolitan regions in the North (Hamburg), West (Frankfurt am Main, Wiesbaden, Mainz), South (Munich, Augsburg), and East (Leipzig) of Germany. A total number of 671 individuals was recruited for the NAPSHI study. Homeless people in lodging houses, night shelters, day shelters, women’s shelters, drug counselling centres, and specialised medical practices were invited to participate. Due to the hard-to-reach nature of the population, a convenience sample was used. Inclusion criteria were age ≥ 18 and duration of homelessness of ≥7 days where only pregnancy was an exclusion criterion. The participants were asked to fill out a standardised questionnaire that was provided in several languages. If required, the interviews were conducted face-to-face or with the help of a translator. A physical examination and a blood withdrawal followed the interview. The present manuscript focuses exclusively on questionnaire-based data. Every participant was offered an incentive of €5 for the procedure, which required approximately 30 min. All participants provided written informed consent before the examination. Approval by the Ethics Committee of the Hamburg Chamber of Physicians was provided (No.: PV7333).

### 2.2. Dependent Variables

In this study, the Patient Health Questionnaire-9 (PHQ-9) was used to measure probable depression, and the Generalized Anxiety Disorder Screening Tool (GAD-2) was used to measure probable anxiety. Both questionnaires are self-reported questionnaires and evaluate symptoms of depression or anxiety during the past 14 days. The PHQ-9 and GAD-2 are validated screening instruments capturing core symptoms of depressive and anxiety disorders. While they do not replace clinical diagnosis, their scores are strongly associated with clinically relevant symptom severity, supporting their use in epidemiological studies among vulnerable populations. Both screening tools are validated for clinical use and research [15,16]. The PHQ-9 is the depression module of the Patient Health Questionnaire that scores each of the 9 DSM-IV criteria for depressive disorders as “0” (not at all), “1” (several days), “2” (more than half of the days) or “3” (nearly every day) [15]. For the PHQ-9, the categorial interpretation was used with the recommended cut-off of 10 points. A sum score of 10 points or more was defined as “depression”, whereas a sum score below 10 points was defined as “no depression” resulting in binary results. For the PHQ-9, a cut-off score of ≥10 has 88% sensitivity and specificity for major depression [15]. The GAD-2 covers two main criteria for generalised anxiety disorders and scores them from “0” (not at all) to “3” (nearly every day). For the GAD-2, the recommended cut-off for probable anxiety was used, defining a score ≥ 3 as “anxiety” and a score < 3 as “no anxiety”. A cut-off score of ≥3 in the GAD-2 has an 86% sensitivity for generalised anxiety disorder and a specificity of 83% [17].

### 2.3. Independent Variables

Independent variables were selected on a clinical basis, which included theoretical assumptions and recent or similar studies and research, such as the “Hamburg Survey of Homeless Individuals” [13]. We included the following independent variables in the analysis: age (18–29; 30–39; 40–49; 50–59; 60–80), sex (male; female), nationality (German; EU; non-EU), region of participation (Hamburg, Frankfurt, Leipzig, München), duration of homelessness (in years), health insurance status (yes; no), level of education (no school education; school education; vocational education; higher tertiary education), marital status (married; single; widowed; divorced), consumption of alcohol or illegal drugs (never; once, twice a year; monthly; weekly; daily), preceding imprisonment (yes; no), and the concern of being infected with COVID-19 (not at all; a little; some; severe). The ETHOS classification was used to distinguish between housing conditions (homeless and roofless) [18].

### 2.4. Statistical Analysis

First, the characteristics of the analytical sample were described. Numbers and percentages were used to describe categorical variables. Continuous variables were inspected for approximate normality using histograms, and medians with interquartile ranges were used to describe continuous variables. The chi-squared test compared expected and observed counts between groups when expected counts yielded above five. A Wilcoxon–Mann–Whitney-U test was used to compare medians between two groups where the underlying distribution was similar and a location shift was a plausible hypothesis. Logistic regression was performed to identify the key predictors of depression and anxiety. Independent variables were included on a clinical basis and based on theoretical considerations, involving results of previous studies like the “Hamburg study on the health of homeless individuals”. Continuous variables were included in their linear functional form without exploring the fit of higher polynomial terms. A complete-case analysis was employed. The statistical size was defined as 0.05 for all statistical tests. The analyses were conducted using Stata 17.0 (StataCorp LLC (College Station, TX, USA)).

## 3. Results

### 3.1. Sample Characteristics and Prevalence of Probable Depression and Anxiety

The total sample included 671 participants. The median age was 43 years (IQR: 35–52). 81.8% (n = 532) of the individuals were male. Further sample characteristics are shown in Table 1. Excluding individuals with incomplete questionnaires, the analytical sample contained 612 participants for the depression analysis and 632 participants for the anxiety analysis. Differences between participants with and without available data on probable depression and probable anxiety are displayed in Supplementary Table S1. Based on the PHQ-9 cut-off values, 26.8% (95% CI: 23.2–30.3 of the individuals had probable depression. We detected 27.2% (95% CI: 23.7–30.7) of the individuals with probable anxiety according to the GAD-2 cut-off values.

In univariable analyses, a significant difference in the prevalence of anxiety between male (25.9%, N = 132) and female (35.1%, N = 40) participants was found, revealing anxiety to be more frequent in females (*p* = 0.05). Likewise, a significant difference in prevalence was seen for depression, with a higher percentage of female (36.4%, N = 40) than male (24.9% N = 123) participants with probable disease (*p* = 0.01). Comparing the prevalence of depression and anxiety by illegal drug consumption pattern, a significant difference in prevalence was observed (depression: *p* < 0.0001; anxiety: *p* = 0.01). Generally, a higher prevalence of depression and anxiety was observed in individuals more frequently consuming illegal drugs: For anxiety, participants with daily drug consumption showed a prevalence of 36.2%, N = 50, while participants who reported never consuming illegal drugs had a lower prevalence of 22.7%, N = 82. For depression, we found a prevalence of 45.4%, N = 60, for daily consumption versus a prevalence of 18.5%, N = 65, for those who stated to never consume illegal drugs. Interestingly, a significant difference in the prevalence of depression was detected between former prisoners and those who reported never having been incarcerated (*p* = 0.001). Participants who reported previous incarceration were found to have a higher prevalence (32.9%, N = 106) of probable depression compared to those who had not been incarcerated previously (20.2%, N = 55). A significant difference in depression prevalence between cities where the interviews were conducted was found (*p* = 0.01), with a higher prevalence of depression in the metropolitan region of Hamburg (34.8%, N = 70) compared to the other three cities with comparable portions (Frankfurt: 21.3%, N = 29; Leipzig: 26.0%, N = 27; Munich: 22.2%, N = 38). When comparing the prevalence of anxiety in participants who reported different levels of COVID-19 fear, moderate evidence for a difference in prevalence was found (*p* = 0.02), with participants who reported a higher fear of COVID-19, exhibiting a higher prevalence of anxiety (some fear: 40.0%, N = 20; strong fear: 36.6%, N = 15) than those who had no fear of COVID-19 (23.3%, N = 101). Further univariable comparisons are displayed in Table 1.

### 3.2. Key Predictors of Probable Depression and Anxiety

In the regression analysis, 520 participants were included for probable depression, and 533 participants were included for probable anxiety. Although displayed in the univariable analysis, the variable “mental health problems as the reason for the continuance of homelessness” was not included in the multivariable regression due to collinearity issues. Findings of multiple logistic regressions are displayed in Table 2.

The multivariable logistic regression analysis showed both probable depression and anxiety associated with the female gender (depression: OR 1.80 [95% CI: 1.06 to 3.03]; anxiety: OR: 1.69 [95% CI: 1.03–2.76]).

Participants who reported daily use of illegal substances had 3.10 (95% CI: 1.87–5.49), and participants reporting a rare use of illegal substances had 2.75 (95% CI: 1.03–7.32) times the odds of probable depression compared to participants who did not use substances. Similarly, participants reporting daily use of illegal substances had 2.04 (95% CI: 1.21–3.45), and participants reporting weekly use of illegal substances had 2.34 (95% CI: 1.13–4.85) times the odds of anxiety compared to participants who reported not using substances. Furthermore, with every year of duration of homelessness, individuals had 1.004 (95% CI: 1.001–1.007) times the odds of depression compared to those with a shorter duration of homelessness. Participants who reported some fear of contracting COVID-19 had 2.52 (95% CI: 1.31–4.83) times the odds of anxiety, whereas participants who reported a little fear of contracting COVID-19 had 1.81 (95% CI: 1.01–3.23) the odds of depression compared to participants who reported no fear of contracting COVID-19.

## 4. Discussion

### 4.1. Main Findings

The NAPSHI study investigated the prevalence and key predictors of probable depression and anxiety in homeless people during the COVID-19 pandemic in four German metropolitan regions. A high prevalence of both probable depression and anxiety among homeless individuals was found, with probable depression in 26.80% (95% CI 23.2–30.3) and probable anxiety in 27.2% (95% CI 23.7–30.7) of the participants. Using multivariable logistic regression analysis, we identified female gender and frequent use of illegal substances as key predictors for depression and anxiety. Depression was found to be statistically significantly associated with a longer duration of homelessness. Furthermore, a strong fear of contracting COVID-19 was associated with increased odds of anxiety.

Low socioeconomic status has been identified to be associated with an increased risk for depression and anxiety [12,19], and mental health problems were shown to be especially frequent in homeless people [20]. A cross-sectional study conducted in Germany in 2021 identified depression in 20.0% and anxiety in 13.4% of the general population [21]. Among homeless individuals, we identify around one-fourth of the participants suffering from probable anxiety and depression. Precisely, the prevalence of depression in homeless individuals was 34.0%, and the prevalence of anxiety was 103.3% higher compared to the general adult population of Germany in the same period. This difference in prevalence highlights the increased burden of depression and anxiety, and thus the high vulnerability of homeless individuals [21].

In the general population, a sharp increase in individuals suffering from depression and anxiety has been noted compared to pre-pandemic prevalence [21,22]. This increase in mental health issues during the course of the COVID-19 pandemic may also have occurred in the population of homeless individuals. This hypothesis is supported by comparing data acquired during the “Hamburg study on the health of homeless individuals” of 2020 with data acquired in Hamburg during the NAPSHI study. Here, homeless people in the region of Hamburg showed a rise from 22.5% to 34.8% in probable depression and a rise from 19.7% to 30.1% in probable anxiety [13]. This rise may be fostered by increased social isolation resulting from COVID-19-related social distancing measures, along with reduced availability of aid facilities for homeless individuals during the progression of the pandemic [23,24]. However, the comparability of both studies is limited due to differences in instruments and sample composition with totally separated cohorts.

Nevertheless, a trend of increasing anxiety and depression in homeless people during the COVID-19 pandemic is also suggested comparing our data with international studies from before the pandemic. A worldwide meta-analysis conducted by Fazel and colleagues found a pooled prevalence of 11.4% (95% CI: 8.4 to 14.4) for major depression in homeless people in 2008, which is less than half compared to studies conducted during the COVID-19 pandemic [25]. For instance, a study applying the PHQ-9 in homeless individuals in France in the Spring of 2020 found that more than half of the individuals showed signs of depression [26]. In line with our data, this study showed an increased prevalence of depression compared to French national averages at the same time, as well as an increased prevalence of depression compared to data acquired in homeless populations pre-pandemic. Key predictors of depression in this study were being female, single, chronically ill, facing food insecurities, and the participant’s region of origin [26].

In regression analysis, we identified female sex as a key determinant for depression and anxiety. Evidence suggests that homeless women are more likely to suffer from mental health difficulties and that the prevalence of depression and anxiety in homeless women may have increased during the COVID-19 pandemic [27,28]. Several international studies confirm that the female sex is a predictor of depression and anxiety [29,30].

We observed a high number of homeless people with probable depression or anxiety reporting frequent alcohol consumption and use of illegal substances versus lower numbers of homeless people with depression or anxiety. The observed association between illegal drug consumption and mental health difficulties might be of reverse causation, and feedback between the exposures might occur, further aggravating health difficulties in people experiencing homelessness [31,32]. In line with this, a Canadian study showed a high prevalence of substance abuse in homeless people and an association of substance abuse with impaired mental health [33]. Interestingly, we observed no evidence of a difference in the odds of depression or anxiety in homeless people reporting different alcohol consumption patterns. As for the consumption of illegal substances, the link between alcohol consumption and homelessness may be bi-directional, and studies show frequent alcohol consumption in individuals suffering from depression [34]. However, compared with other countries, alcohol consumption among homeless people in Germany is more frequent, possibly as a consequence of easier affordability and accessibility of alcohol in Germany [34,35].

In addition, a proportion of participants reported neither substance use nor mental health problems as a reason for the continuation of homelessness. This underlines the heterogeneity of the homeless population. Qualitative interviews of these individuals may reveal structural, actionable factors that should be considered in public health interventions.

In line with this, findings from a comparable cross-sectional study among undocumented migrants similarly indicate that mental health outcomes are strongly shaped by structural and social conditions rather than individual pathology alone [36]. Qualitative research further suggests that social integration and supportive environments are critical determinants of recovery for homeless individuals with serious mental illness [37].

In our study, more than half of the participants reported previous incarceration. Furthermore, around two-thirds of the individuals we identified with probable depression had been in prison before. However, former imprisonment was not identified as a key determinant in multivariable analysis, possibly due to low sample sizes as indicated by wide confidence intervals. In the literature, the association between depression and incarceration has previously been highlighted by a Canadian study, concluding that former prisoners are more likely to suffer from depression, which underlines the relevance of psychological support offered in public prisons and beyond [38].

The link between the outcome of housing provision and mental health is well described. Studies show housing provision with mental health support to be superior to mental health care alone and mental health care to be more effective when provided by specialised healthcare organisations [39]. Housing has, furthermore, been confirmed to improve the health of homeless individuals with anxiety and depression [40]. In line with this, we found a higher prevalence of depression and anxiety in homeless who are roofless than those who are homeless according to the ETHOS classification (58.1% vs. 41.9% for depression and 51.2% vs. 48.8% for anxiety). Targeting this, the European Pillar of Social Rights Action Plan has set housing and housing assistance as a fundamental principle [41]. The German Federal government published a National Action Plan to defeat homelessness by 2030 in Mai 2024, presenting measures for housing of homeless people, health and mental health improvement as well as prevention and research in homelessness [42].

### 4.2. Strengths and Limitations

The NAPSHI Study is the first multicentre study of the physical and mental health of homeless individuals in Germany [43]. The homeless population is a fluctuating, vulnerable, and heterogeneous group of individuals that is difficult to access. We included a large number of participants in a variety of places with different social or medical services. However, homeless individuals without contact with aid facilities were not included, which may have caused selection bias. The utilised screening tools, the PHQ-9 and the GAD-2 are well-established tools for depression and anxiety. In the German questionnaire, validated translations of PHQ-9 and GAD-2 were used. Our study is cross-sectional, thus the influence of the COVID-19 pandemic on anxiety and depression cannot be quantified and a longitudinal sample is required. Furthermore, qualitative research is necessary to better examine the relationship between homelessness and depression or anxiety.

## 5. Conclusions

This study highlights the high prevalence of depression and anxiety in the homeless population in Germany. Our data may help identify at-risk homeless individuals who may benefit from targeted intervention. Given the reciprocal connection between housing and mental health problems, housing, as well as treatment of mental illnesses, must be focused on equally when addressing homelessness. Longitudinal studies are required to confirm increased prevalence due to pandemic measures.

## Figures and Tables

**Table 1:** Sample characteristics.

	Total Sample N (%) or Median (IQR)	Probable Depression (PHQ-9 ≥ 10) N (%) or Median (IQR)	*p*-Value	Probable Anxiety (GAD-2 ≥ 3) N (%) or Median (IQR)	*p*-Value
**Overall**	N = 671	N = 164/612 (26.80%)		N = 172/632 (27.22%)	
**Age, years**			0.015 *		0.270
18–29	85 (13.5)	24 (15.2)		27 (16.5)	
30–39	169 (26.8)	53 (33.5)		46 (28.0)	
40–49	166 (26.3)	44 (27.8)		40 (24.4)	
50–59	152 (24.1)	25 (15.8)		32 (19.5)	
60–80	59 (9.4)	12 (7.6)		19 (11.6)	
**Sex**			0.014 *		0.047 *
Male	532 (81.8)	123 (75.5)		132 (76.7)	
Female	118 (18.2)	40 (24.5)		40 (23.3)	
**Country of birth**			0.200		0.268
Germany	362 (59.8)	101 (67.8)		104 (66.2)	
EU country	197 (32.6)	38 (25.5)		41 (26.1)	
No-EU country	46 (7.6)	10 (6.7)		12 (7.6)	
**ETHOS-Classification**			0.763		0.076
Roofless	353 (57.3)	93 (58.1)		85 (51.2)	
Houseless	263 (42.7)	67 (41.9)		81 (48.8)	
**Duration of homelessness, years**	1.5 (0.42–4)	2 (0.5–6)	0.039 *	1.33 (0.42–5)	0.569
**Health insurance status**			0.600		0.147
No health insurance	199 (31.4)	47 (29.9)		45 (27.3)	
Health insurance	435 (68.6)	110 (70.1)		120 (72.7)	
**Level of Education**			0.111		0.275
No school education	113 (18.1)	36 (22.9)		31 (18.7)	
School education	287 (46.0)	74 (47.1)		85 (51.2)	
Vocational education	178 (28.5)	40 (25.5)		41 (24.7)	
Higher tertiary education	46 (7.4)	7 (4.5)		9 (5.4)	
**Marital Status**			0.651		0.577
Married	74 (11.8)	19 (12.0)		20 (12.0)	
Single	421 (67.0)	111 (70.3)		117 (70.5)	
Widowed	17 (2.7)	4 (2.5)		3 (1.8)	
Divorced	116 (18.5)	24 (15.2)		26 (15.7)	
**Alcohol consumption**			0.150		0.145
Never	213 (33.8)	49 (30.6)		48 (29.6)	
Once to twice a year	67 (10.6)	15 (9.4)		15 (9.3)	
Monthly	83 (13.2)	22 (13.8)		23 (14.2)	
Weekly	79 (12.5)	16 (10.0)		16 (9.9%)	
Daily	188 (29.8)	58 (36.3)		60 (37.0)	
**Use of illegal substances**			<0.001 *		0.011 *
Never	375 (59.3)	65 (40.4)		82 (48.5)	
Once to twice a year	22 (3.5)	8 (5.0)		7 (4.1)	
Monthly	50 (7.9)	14 (8.7)		12 (7.1)	
Weekly	47 (7.4)	14 (8.7)		18 (10.7)	
Daily	138 (21.8)	60 (37.3)		50 (29.6)	
**Mental health problems as reason for continuance of homelessness**			<0.001 *		<0.001 *
No	528 (86.7)	118 (73.8)		126 (75.9)	
Yes	81 (13.3)	42 (26.3)		40 (24.1)	
**Former imprisonment**			0.001 *		0.674
No	287 (45.8)	55 (34.2)		74 (44.0)	
Yes	340 (54.2)	106 (65.8)		94 (56.0)	
**Fear of contracting COVID-19**			0.118		0.021 *
Not at all	440 (71.0)	100 (64.1)		101 (62.3)	
A bit	87 (14.0)	28 (17.9)		26 (16.0)	
Some	52 (8.4)	13 (8.3)		20 (12.3)	
Strongly	41 (6.6)	15 (9.6)		15 (9.3)	
**City of data acquisition**			0.014 *		0.674
Hamburg	212 (31.6)	70 (42.7)		62 (36.0)	
Frankfurt	153 (22.8)	29 (17.7)		36 (20.9)	
Leipzig	108 (16.1)	27 (16.5)		29 (16.9)	
München	198 (29.5)	38 (23.2)		45 (26.2)	

Baseline characteristics of the cohort stratified by anxiety (defined by Generalized Anxiety Disorder Screening Tool (GAD-2) >2) and depression (defined by Patient Health Questionnaire 9 > 10). Data are presented as n (%) or median (IQR), based on the number of valid observations for each variable. Variations in sample sizes across variables are due to missing data. * Variables marked with an asterisk are significantly associated with the respective outcome in univariable analyses (*p* < 0.05). A chi-squared test compared expected and observed numbers across groups when numbers yielded above five. Wilcoxon–Mann–Whitney-U test was used to compare medians between two groups where the underlying distribution was similar. The statistical size was defined as 0.05 for all statistical tests. Abbreviation: IQR, interquartile range; EU, European Union; ETHOS, European Typology of Homelessness and Housing Exclusion; COVID-19, Coronavirus 2019 Disease.

**Table 2:** Determinants of probable depression and anxiety.

Variables	Depression	Anxiety
Sex (reference group: male)		
	1.80 *	1.69 *
Age category (reference group: 18 to 29 years)	(1.06–3.03)	(1.03–2.76)
>30–39 years	1.09	0.68
	(0.57–2.08)	(0.36–1.27)
>40–49 years	0.77	0.61
	(0.39–1.52)	(0.32–1.16)
>50–59 years	0.56	0.63
	(0.27–1.18)	(0.32–1.23)
>60 years	1.02	1.28
	(0.41–2.54)	(0.57–2.86)
Duration of homelessness (years)	1.004 *	1.00
	(1.001–1.007)	(1.00–1.00)
Use of illegal substances (reference group: no use of illegal substances)		
once, twice a year	2.75 *	1.75
	(1.03–7.32)	(0.64–4.76)
monthly	1.76	1.16
	(0.84–3.69)	(0.55–2.46)
weekly	1.86	2.34 *
	(0.84–4.14)	(1.13–4.85)
daily	3.20 ***	2.04 **
	(1.87–5.49)	(1.21–3.45)
Former imprisonment (reference group: no former imprisonment)		
yes	1.38	1.11
	(0.85–2.26)	(0.70–1.74)
Fear of contracting COVID-19 (reference group: not at all)		
A little	1.81 *	1.42
	(1.01–3.23)	(0.81–2.50)
some	1.43	2.52 **
	(0.68–3.01)	(1.31–4.83)
severe	1.94	1.88 ^+^
	(0.87–4.33)	(0.89–4.00)
Constant	0.14 ***	0.28 ***
	(0.08–0.27)	(0.16–0.50)
Observations	520	533
Pseudo R-squared	0.10	0.05

Odds ratios are reported; 95% confidence intervals are in parentheses. *** *p* < 0.001, ** *p* < 0.01, * *p* < 0.05, ^+^ *p* < 0.10. Table 2: Logistic regression analysis of anxiety (defined by Generalized Anxiety Disorder Screening Tool (GAD-2) > 2) and depression (defined by Patient Health Questionnaire 9 (PHQ9) > 10). Independent variables were included on a clinical basis. The statistical significance was defined as 0.05 for all statistical tests. Abbreviation: COVID-19, Coronavirus-Disease 2019.

## Data Availability

The original contributions presented in this study are included in the article/Supplementary Material. Further inquiries can be directed to the corresponding author.

## Supplementary Materials

The following supporting information can be downloaded at: https://www.mdpi.com/article/10.3390/ijerph23020154/s1, Table S1: Baseline characteristics of the cohort stratified by missing data for probable anxiety/probable depression. Abbreviation: IQR, interquartile range; EU, European Union; ETHOS, European Typology of Homelessness and Housing Exclusion; COVID-19, Coronavirus 2019 Disease.
