# Supplementary material for: Prevalence and Correlates of Probable Depression and Anxiety Among Homeless Individuals During the COVID-19 Pandemic in Germany
Source: Int J Environ Res Public Health. 2026 Jan 26;23(2):154. doi: 10.3390/ijerph23020154 (PMC12941050; doi:10.3390/ijerph23020154)
Supplement: Supplementary file 1 [file ijerph-23-00154-s001.zip › ijerph-3982746-supplementary.pdf]

Supplements

Table S1

	No missing values for probable anxiety N(%) or Median (IQR)	Missing values for probable anxiety N(%) or Median (IQR)	No missing values for probable depression N(%) or Median (IQR)	Missing values for probable depression N(%) or Median (IQR)
**Overall**	N=632	N=39	N=612	N=59
**Age, years**				
18-29	83 (13.7)	3 (13.0)	82 (13.9)	4 (9.3)
30-39	161 (26.5)	8 (34.8)	155 (26.4)	14 (32.6)
40-49	158 (26.0)	8 (34.8)	152 (25.9)	14 (32.6)
50-59	148 (24.3)	3 (13.0)	142 (24.1)	9 (20.9)
60-80	58 (9.5)	1 (4.3)	57 (9.7)	2 (4.7)
**Sex**				
Male	510 (81.7)	22 (84.6)	494 (81.8)	38 (82.6)
Female	114 (18.3)	4 (15.4)	110 (18.2)	8 (17.4)
**Country of birth**				
Germany	358 (61.4)	4 (18.2)	349 (61.8)	13 (32.5)
EU country	181 (31.0)	15 (68.2)	174 (30.8)	22 (55.0)
No-EU counrty	44 (7.5)	3 (13.6)	42 (7.4)	5 (12.5)
**ETHOS-Classification**				
Roofless	341 (56.9)	11 (68.8)	332 (57.0)	20 (60.6)
Houseless	258 (43.1)	5 (31.3)	250 (43.0)	13 (39.4)
**Duration of homelessness, years**	1,5 (0,4-4)	0,75 (0,2-1,6)	1,5 (0,4-4)	1,17 (0,5-3)
**Health insurance status**				
No health insurance	195 (31.6)	3 (17.6)	188 (31.4)	10 (27.8)
Health insurance	422 (68.4)	14 (82.4)	410 (68.6)	26 (72.2)
**Level of education**				
No school education	110 (18.0)	3 (20.0)	107 (18.1)	6 (18.2)
School education	280 (45.9)	7 (46.7)	272 (45.9)	15 (45.5)
Vocational education	174 (28.5)	5 (33.3)	169 (28.5)	10 (30.3)
Higher tertiary education	46 (7.5)		44 (7.4)	2 (6.1)
**Marital status**				
Married	70 (11.5)	4 (22.2)	64 (10.8)	10 (27.8)
Single	411 (67.4)	10 (55.6)	404 (68.2)	17 (47.2)
Widowed	16 (2.6)	1 (5.6)	15 (2.5)	2 (5.6)
Divorced	113 (18.5)	3 (16.7)	109 (18.4)	7 (19.4)
**Alcohol consumption**				
Never	206 (33.6)	7 (43.8)	203 (33.9)	10 (31.3)
Once, twice a year	67 (10.9)		67 (11.2)	
Monthly	81 (13.2)	2 (12.5)	81 (13.5)	2 (6.3)
Weekly	77 (12.5)	2 (12.5)	75 (12.5)	4 (12.5)
Daily	183 (29.8)	5 (31.3)	172 (28.8)	16 (50.0)
**Abuse of illegal substances**				
Never	361 (58.7)	14 (82.4)	352 (58.9)	23 (67.6)
Once, twice a year	21 (3.4)	1 (5.9)	22 (3.7)	
Monthly	49 (8.0)	1 (5.9)	48 (8.0)	2 (5.9)
Weekly	46 (7.5)	1 (5.9)	44 (7.4)	3 (8.8)
Daily	138 (22.4)		132 (22.1)	6 (17.6)
**Mental health problems as reason for continuance of homelessness**				
No	515 (86.6)	13 (92.9)	501 (86.8)	27 (84.4)
Yes	80 (13.4)	1 (7.1)	76 (13.2)	5 (15.6)
**Former imprisonment**				
No	278 (45.5)	9 (60.0)	273 (45.9)	14 (45.2)
Yes	333 (54.5)	6 (40.0)	322 (54.1)	17 (54.8)

**Fear of contracting COVID-19**				
Not at all	433 (71.1)	7 (63.6)	421 (71.1)	19 (67.9)
A bit	85 (14.0)	2 (18.2)	84 (14.2)	3 (10.7)
Some	50 (8.2)	2 (18.2)	46 (7.8)	6 (21.4)
Strongly	41 (6.7)		41 (6.9)	
**City of data acquisition**				
Hamburg	206 (32.6)	6 (15.4)	201 (32.8)	11 (18.6)
Frankfurt	140 (22.2)	13 (33.3)	136 (22.2)	17 (28.8)
Leipzig	105 (16.6)	3 (7.7)	104 (17.0)	4 (6.8)
München	181 (28.6)	17 (43.6)	171 (27.9)	27 (45.8)

Table S1: Baseline characteristics of the cohort stratified by missing data for probable anxiety/probable depression. Abbreviation: IQR, interquartile range; EU, European Union; ETHOS, European Typology of Homelessness and Housing Exclusion; COVID-19, Coronavirus 2019 Disease
